# Prevalence of psychological distress in elderly hypertension patients in primary care

**DOI:** 10.1007/s12471-013-0502-z

**Published:** 2013-12-05

**Authors:** L. Ringoir, S. S. Pedersen, J. W. M. G. Widdershoven, V. J. M. Pop

**Affiliations:** 1 Department of Medical and Clinical Psychology, Centre of Research on Psychology in Somatic Diseases (CoRPS), Tilburg University, PO Box: 90153, 5000 LE Tilburg, the Netherlands; 2 Department of Cardiology, TweeSteden Hospital, Dr. Deelenlaan 5, 5042 AD Tilburg, the Netherlands; 3 Department of Cardiology, Thoraxcenter, Erasmus Medical Centre, Rotterdam, the Netherlands; 4 Department of Cardiology, Odense University Hospital, Odense, Denmark; 5 Institute of Psychology, University of Southern Denmark, Odense, Denmark

**Keywords:** Hypertension, Psychological distress, Primary care, Depression, Anxiety, Type D personality

## Abstract

**Background:**

Recent guidelines on cardiovascular disease prevention advocate the importance of psychological risk factors, as they contribute to the risk of developing cardiovascular disease. However, most previous research on psychological distress and cardiovascular factors has focused on selected populations with cardiovascular disease.

**Aim:**

The primary aim was to determine the prevalence of depression, anxiety, and Type D personality in elderly primary care patients with hypertension. Secondary aim was to examine the relation between elevated systolic blood pressure and depression, anxiety, and Type D personality.

**Design and Setting:**

A cross-sectional study in primary care practices located in the south of the Netherlands.

**Method:**

Primary care hypertension patients (*N* = 605), between 60 and 85 years (45 % men, mean age = 70 ± 6.6), were recruited for this study. All patients underwent a structured interview including validated self-report questionnaires to assess depression (PHQ-9), anxiety (GAD-7), and Type D personality (DS14) as well as blood pressure assessment.

**Results and Conclusion:**

Depression was prevalent in 5 %, anxiety in 5 %, and Type D personality in 8 %. None of the distress measures were associated with elevated systolic blood pressure of >160 mmHg (all *p*-values >0.05). This study showed no relation between psychological distress and elevated systolic blood pressure in elderly primary care patients with hypertension.

## Introduction

Hypertension is an important risk factor for cardiovascular disease, such as coronary artery disease (CAD), heart failure and stroke [1] with the mean prevalence of hypertension in six European countries being 44 % (50 % in men, 39 % in women) [2]. Systolic blood pressure increases with age, with the mean systolic blood pressure (SBP) of European citizens aged 60–64 years exceeding 140 mmHg, which increases to a mean SBP of 150 mmHg for those aged 70–74 years [2].

One of the key messages in the 2012 European guidelines on cardiovascular disease prevention in clinical practice is that assessment of psychosocial factors such as depression, anxiety, and Type D personality (the tendency to experience negative emotions combined with the tendency to inhibit expression of emotions [3]) in patients with cardiovascular disease (CVD) risk factors is crucial, as they contribute to the risk of developing CVD [4]. However, more recent and larger studies have reported smaller or no effects of Type D personality on cardiovascular outcomes or mortality as compared with earlier studies [5]. A recent systematic review showed an increased incidence of hypertension in individuals with elevated symptoms of depression [6]. In contrast with the European guidelines [4], and cardiac rehabilitation where screening for anxiety and/or depression is recommended [7], psychological factors are not included in the multifactorial [8] Dutch primary care guidelines on Cardiovascular Risk Management [9].

In patients with hypertension, psychological distress may serve as a barrier against adequate medication adherence [10]. Prior findings have shown an independent association between poor medication adherence in elderly patients with hypertension and depression and anxiety [10, 11]. In general, adherence is considered paramount for the successful treatment of hypertension, with non-adherence as one of the possible causes of resistant hypertension [12].

The objectives of the current study were to examine (1) the prevalence of symptoms of depression and anxiety and Type D personality in elderly primary care patients with hypertension, and (2) the relation between elevated symptoms of depression, anxiety, Type D personality, and systolic blood pressure.

## Methods

### Participants and design

Between June 2010 and January 2012, primary care patients aged between 60 and 85 years, with diagnosed hypertension according to their medical records, were recruited for this cross-sectional study from five general practices affiliated with the primary care organisation PoZoB. Exclusion criteria were: Previous diagnosis of heart failure; current treatment by a cardiologist; history of severe psychiatric illness other than mood or anxiety disorders; cognitive impairments; terminal cancer; insufficient knowledge of the Dutch language; illiteracy or inability to read due to visual impairments. The study protocol was approved by the medical ethics committee of the Elisabeth Hospital, Tilburg, the Netherlands. All participants provided informed consent.

### Measures

#### Demographic and clinical variables

Demographic variables assessed during a structured interview at the local GP office included age, gender, education level, and marital status. Clinical variables assessed during the structured interview included height, weight, blood pressure (after 20 and 40 min of resting), current smoking, alcohol consumption, current or past depression, and current or past anxiety. Information on clinical variables obtained via chart extraction and using ICPC codes were previous myocardial infarction, peripheral arterial disease, cerebrovascular accident or transient ischaemic attack (CVA/TIA), chronic obstructive pulmonary disease (COPD), type 2 diabetes, and years since diagnosis of hypertension.

#### Psychological measures

Psychological distress was assessed with patient-report questionnaires which were filled out during the structured interview. The 14-item Type D Scale (DS14) was used to assess Type D personality with 7 items tapping into negative affectivity (NA) and 7 items into social inhibition (SI). Items are rated on a 5-point Likert scale. The SI and NA scales can be used as continuous scales (range 0–28), and also to classify patients as ‘Type D’ versus ‘non-Type D’, using a cut-off of 10 on both NA and SI that was found to be optimal according to item response theory [13]. The DS14 is a valid and reliable instrument to assess Type D personality [14]. Symptoms of depression were measured with the Patient Health Questionnaire-9 (PHQ-9). The nine items are rated on a 4-point Likert scale (range 0–27). For this study a cut off of ≥9 was used, since it is considered suitable for elderly individuals in primary care, with a sensitivity of 0.88 and specificity of 0.80, and an area under the curve for the detection of a major depressive disorder of 0.87 [15]. Symptoms of anxiety were measured with the Generalized Anxiety Disorder-7 (GAD-7) scale. The seven items are rated on a 4-point Likert scale (range 0–21). A cut-off of ≥8 has a high sensitivity and specificity for the detection of generalised anxiety disorder (sensitivity = 0.92 and specificity = 0.76) as well as for the detection of any anxiety disorder (sensitivity = 0.77 and specificity = 0.82), the area under the curve for detecting generalised anxiety disorder is 0.91 [16].

Both the PHQ-9 and the GAD-7 are derived from the Primary Care Evaluation of Mental Disorders (PRIME-MD), which was originally designed for the diagnosis of five mental disorders (depression, anxiety, alcohol abuse, somatoform disorder, and eating disorder) in the primary care setting by using DSM IV criteria [17].

### Statistical analyses

Statistical analyses were performed using IBM Statistical Package for the Social Sciences version 18.0. Because previous research has shown differences in prevalence of depression and anxiety between men and women, the different psychological indices are reported for the total group and for men and women separately [18]. Student’s t-tests or Welch’s t-tests (two-tailed) were used to assess differences in mean scores between groups when appropriate. Unadjusted odds ratios were calculated using univariable logistic regression analysis, with an elevated systolic blood pressure >160 mmHg as dependent variable which was calculated using the mean of the two blood pressure assessments. Adjusted odds ratios were calculated using multiple logistic regression, also with an elevated systolic blood pressure >160 mmHg as dependent variable and elevated symptoms of depression, anxiety, and Type D personality, as independent variables, adjusting for age, gender, having a partner, education, current smoking, alcohol consumption, and BMI.

## Results

**Fig. 1:**
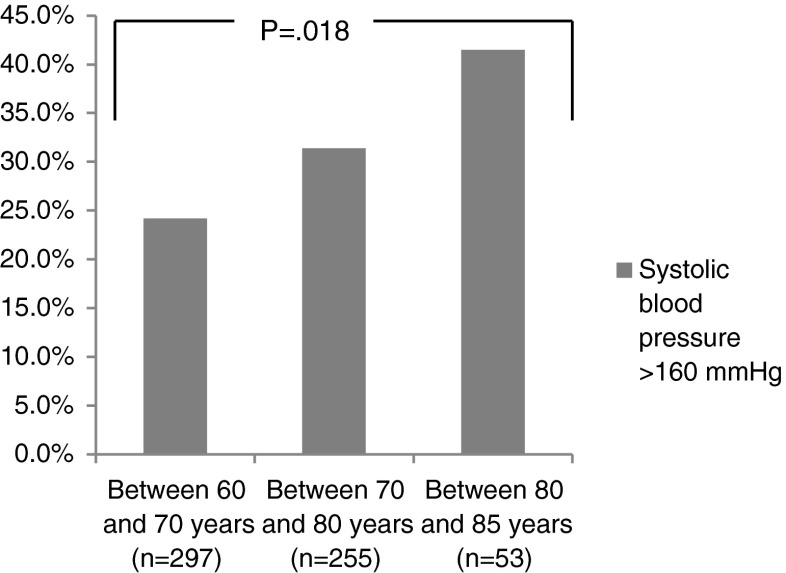
Elevated systolic blood pressure in three age categories

Of the 913 patients with hypertension approached, 619 (68 %) agreed to participate. Fourteen patients were excluded post-hoc as they met the exclusion criterion of being treated by a cardiologist (*n* = 2), missing all PHQ-9, GAD-7, and DS14 scores (*n* = 1), and insufficient knowledge of the Dutch language (*n* = 11). This resulted in 605 patients (66 %) who could be included in the analyses. The baseline characteristics for the total sample and stratified by systolic blood pressure ≤160 mmHg and >160 mmHg, are shown in Table 1. The mean age was 70 years (±6.6) and 45 % of the study population was male. An elevated systolic blood pressure of >160 mmHg was found in 29 % of the patients (*n* = 174). A systolic blood pressure of >160 mmHg was associated with higher age (*p* = 0.006), regular alcohol consumption (*p* = 0.006), and an elevated diastolic blood pressure of >90 mmHg (*p* < 0.001). Using the age categories 60–70 years, 70–80 years, and 80–85 years, prevalence of elevated systolic blood pressure increased significantly with age (*p* = 0.018) (Fig. 1). The proportion of males and females was similar in both participants and non-participants. Women who refused participation were significantly older (*p* < 0.001).

**Table 1:** Baseline characteristics for the total sample and stratified by systolic blood pressure

Characteristic	Total sample	SBP ≤160	SBP >160	p*
	(*n* = 605)	(*n* = 431)	(*n* = 174)	
Demographics				
Male	270 (45 %)	186 (43 %)	84 (48 %)	0.251
**Age (mean ± SD)**	**70.0 (6.6)**	**69.5 (6.5)**	**71.1 (6.5)**	**0.006**
Having a partner	455 (75 %)	323 (75 %)	132 (76 %)	0.812
Low educational level	78 (13 %)	50 (12 %)	28 (16 %)	0.136
Risk factors				
Smoking	81 (13 %)	55 (13 %)	26 (15 %)	0.476
**Alcohol consumption ≥2 glasses/day (on average)**	**194 (32 %)**	**124 (29 %)**	**70 (40 %)**	**0.006**
BMI kg/m^2^ (mean ± SD, *n* = 600)	28.0 (4.4)	28.1 (4.5)	27.9 (4.2)	0.628
Medical history				
Years since diagnosis (mean ± SD)	12.3 (11.2)	11.8 (10.2)	013.6 (13.1)	0.105
**Diastolic blood pressure >90 mm/Hg**	**126 (21 %)**	**52 (12 %)**	**74 (43 %)**	**<0.001**
Previous myocardial infarction	27 (5 %)	20 (5 %)	7 (4 %)	0.739
Peripheral arterial disease	23 (4 %)	15 (4 %)	8 (5 %)	0.515
TIA/stroke	55 (9 %)	38 (9 %)	17 (10 %)	0.712
Diabetes type II	68 (11 %)	52 (12 %)	16 (9 %)	0.312

Bold entries significance/alpha level is 0.05

As shown in Table 2, the prevalence of depression using a cut-off score of ≥9 on the PHQ-9 was 5 %, for anxiety using a cut-off of ≥8 on the GAD-7 this was 5 %, and for Type D personality 8 % when using a cut-off of ≥10 on both the negative affectivity and social inhibition subscales of the DS14. Women had significantly higher mean depression, anxiety, and negative affect scores than men.

**Table 2:** Prevalence and mean scores of depression, anxiety, and Type D in 605 elderly primary care hypertension patients stratified by gender

	Total sample	Men	Women	p*
Prevalence				
**Depression (PHQ-9 ≥9)**	**30 (5 %)**	**6 (2 %)**	**24 (7 %)**	**0.006**
Anxiety (GAD-7 ≥8)	31 (5 %)	11 (4 %)	20 (6 %)	0.293
**Type D personality** ^*^	**49 (8 %)**	**15 (6 %)**	**34 (10 %)**	**0.041**
Mean scores				
**Depression symptoms (PHQ-9 total score)**	**2.2 (3.0)**	**1.5 (2.4)**	**2.7 (3.4)**	**<0.001**
**Anxiety symptoms (GAD-7 total score)**	**1.9 (2.9)**	**1.4 (2.6)**	**2.2 (3.1)**	**0.001**
**DS14 negative affect**	**4.3 (4.8)**	**3.2 (4.3)**	**5.1 (5.1)**	**<0.001**
DS14 social inhibition	6.0 (5.7)	5.7 (5.3)	6.3 (5.9)	0.207

*A score of ≥10 on both the negative affect and social inhibition subscales

In unadjusted analysis (Table 3), age (OR = 1.038, 95 % CI = 1.010–1.066) and alcohol consumption ≥2 glasses a day (OR = 1.666, 95 % CI = 1.154–2.407) were significantly associated with a systolic blood pressure of >160 mmHg. Depression, anxiety and Type D personality were not related to high systolic blood pressure. Adjusted logistic regression showed that age (OR = 1.042, 95 % CI = 1.011–1.073) and regular alcohol consumption (OR = 1.661, 1.121–2.426) were significantly related to a systolic blood pressure of >160 mmHg (Table 3). Again there was no association with depression, anxiety, and Type D personality, adjusting for age, gender, marital status, low education, current smoking, regular alcohol consumption, and BMI.

**Table 3:** Associates of systolic blood pressure of >160 mmHg in elderly primary care hypertension patients (unadjusted and adjusted analyses)

	OR	95 % CI
Unadjusted analysis		
Female gender	0.813	0.571–1.158
**Age**	**1.038**	**1.010–1.066**
Having a partner	1.051	0.697–1.583
Low education	1.461	0.886–2.410
Current smoking	1.201	0.726–1.988
**Alcohol consumption ≥ 2 glasses a day (on average)**	**1.666**	**1.154–2.407**
BMI	0.990	0.951–1.031
Type D personality^a^	1.347	0.727–2.495
Depression (PHQ-9 ≥9)	0.907	0.396–2.078
Anxiety (GAD-7 ≥8)	0.855	0.375–1.950
Adjusted analysis		
Female gender	0.879	0.598–1.293
**Age**	**1.042**	**1.011–1.073**
Having a partner	1.300	0.821–2.059
Low education	1.425	0.837–2.428
Current smoking	1.416	0.826–2.428
**Alcohol consumption ≥ 2 glasses a day (on average)**	**1.661**	**1.121–2.462**
BMI	0.999	0.958–1.041
Type D personality^a^	1.563	0.805–3.038
Depression (PHQ-9 ≥9)	0.950	0.370–2.437
Anxiety (GAD-7 ≥8)	0.863	0.339–2.196

^a^A score of ≥10 on both the negative affect and social inhibition subscales

## Discussion

The results of this study indicate a low prevalence of psychological distress in an unselected sample of elderly primary care hypertension patients. Elevated symptoms of depression were found in 5 %, elevated symptoms of anxiety in 5 %, and Type D personality was prevalent in 8 % of the study population. We found no association between elevated symptoms of depression, anxiety, Type D personality and an elevated systolic blood pressure.

The prevalence of depression in the current study is in line with a large study on depression in later life in which the prevalence of depression in adults aged between 55 and 85 years was 2 % for major depressive disorder and 13 % for minor depression [19]. In general up to 50 % of patients with moderate to high scores on the PHQ-9 do have a major depression [20], which would imply that 2.5 % of the patients in the current study could have a major depression. A review on generalised anxiety disorder in primary care has indicated a prevalence between 3 % and 9 %, which is similar to our findings [21]. Results of a population-based study showed a decline in the prevalence of depression, anxiety, and Type D personality with higher age. This is in line with our results on the prevalence of depression and anxiety, however, Type D personality was prevalent in 17 % of their study population of elderly individuals which is more than twice as much compared with our study [22]. Another previous study on Type D personality, also in primary care hypertension patients with a mean age of 60, found a very high prevalence of Type D personality (53 %) [14]. This is in contrast to our findings of a Type D prevalence rate of only 8 %. Another Dutch population-based study showed a prevalence of Type D personality of 13 % [23], which is more in line with the results of our study, indicating that prevalence rates of Type D personality may vary markedly amongst different studies and populations.

The 2012 European guidelines on cardiovascular disease prevention underline the importance of psychological factors in the risk of developing cardiovascular disease [3, 4]. However, there is no evidence that depression treatment reduces cardiovascular events [24], and there is an ongoing debate about the added value of screening for depression in patients with CVD [24, 25]. Nevertheless, depression in patients with cardiovascular disease is not only associated with clinical outcomes, but also with adherence and poorer health status and impairments to quality of life independent of disease severity [26].

The association between hypertension and distress found in previous research could be explained by decreased adherence to antihypertensive medication [10, 11]. However, although as many as 28.9 % of the patients in the current study had an elevated systolic blood pressure of >160 mmHg, there was no significant association with depression, anxiety, and Type D personality. These findings suggest that other factors contribute to poor blood pressure control in elderly patients with hypertension, at least in primary care. The current findings are in line with the results from Friedman et al., who demonstrated no significant difference in depression and anxiety between normotensive and mildly hypertensive participants [27]. Furthermore, the relatively low prevalence of depression in the current study as well as the lack of an association with elevated systolic blood pressure, is also in accordance with the findings of Wiehe et al. who also did not find a higher prevalence of hypertension in depressive individuals [28].

A major strength of this study is the relatively large sample size of an unselected, elderly hypertension cohort in the primary care setting. The high prevalence of hypertension indicates that (elderly) hypertension patients represent a large proportion of primary care patients where cardiovascular risk management is recommended [2].

A limitation of the study is the cross-sectional design, which does not allow to draw conclusions about causation or to evaluate symptoms over time. Furthermore, there were only limited data available on patients who refused participation. It is possible that patients who refused participation were more likely to be depressed and anxious, and more likely to have a Type D personality [29]. Furthermore, the Type D construct as a prognostic indicator for cardiovascular outcomes is the subject of some debate [30]. Initially published studies have reported higher odds ratios for Type D personality as a predictor of mortality and cardiovascular outcomes compared with more recent studies in which more events were reported, which could possibly imply an overestimation of the effect of Type D personality on mortality or cardiovascular events [5]. Finally, depression and anxiety were measured by means of self-report measures rather than a clinical diagnostic interview. However, the PHQ-9 and the GAD-7 are highly validated instruments with good psychometric properties [13–16], and self-reported symptoms of depression and anxiety have been shown to predict cardiac morbidity and mortality in cardiac populations [31, 32].

In conclusion, a low prevalence of symptoms of anxiety, symptoms of depression, and Type D personality was found in the current study. Furthermore, this study shows no relation between depression, anxiety, and Type D personality and an elevated systolic blood pressure. Therefore, prospective research in primary care populations with hypertension is needed to evaluate the evolvement of psychological distress in association with hypertension over time, and to study the association between psychological distress, hypertension, and long-term cardiovascular outcomes.
